# Case Report: Diagnosis of vertebral alveolar echinococcosis upon next-generation sequencing in a suspected tuberculosis

**DOI:** 10.3389/fsurg.2022.984640

**Published:** 2022-09-29

**Authors:** Tiange Song, Shengkun Peng, Xiaoli Zhou, Li Jiang, Jie Zhang

**Affiliations:** ^1^ Department of Laboratory Medicine, Sichuan Provincial People's Hospital, University of Electronic Science and Technology of China, Chengdu, China; ^2^ Sichuan Provincial Key Laboratory for Human Disease Gene Study, Sichuan Provincial People's Hospital, University of Electronic Science and Technology of China, Chengdu, China; ^3^ Research Unit for Blindness Prevention of Chinese Academy of Medical Sciences (2019RU026), Sichuan Academy of Medical Sciences, Chengdu, China; ^4^ Department of Radiology, Sichuan Provincial People's Hospital, University of Electronic Science and Technology of China, Chengdu, China

**Keywords:** next-generation sequencing, diagnosis, alveolar echinococcosis, tuberculosis, case report

## Abstract

**Introduction:**

Alveolar echinococcosis (AE), caused by larval stages of *Echinococcus multilocularis*, is a rare zoonotic disease that mainly involves the liver. The diagnosis of extrahepatic AE is usually difficult. Here, we describe a rare case of vertebral alveolar echinococcosis with a suspected history of spinal tuberculosis, diagnosed by metagenomic next-generation sequencing (mNGS).

**Case Presentation:**

A 44-year-old woman presented with repetitive neck and back pain, with a surgical history of suspected spinal tuberculosis. Magnetic resonance imaging (MRI) showed cystic masses in the craniocervical junction region and effusion around lumbar vertebrae. Multiple culture tests were performed to detect tuberculosis and other pathogens through puncture of the effusion and of cerebrospinal fluid, but the results were all negative. Finally, mNGS of the effusion fluid was performed and *Echinococcus multilocularis* were detected. The results were further confirmed by Sanger sequencing.

**Conclusion:**

This case emphasizes a role of mNGS in the diagnosis of infectious diseases with unknown pathogen. As a newly emerged sensitive and accurate diagnostic strategy, mNGS provides clinicians an opportunity to clarify pathogens in complicated infectious cases, especially in patients with a history of multiple infections.

## Introduction

Echinococcosis represents a parasitic disease caused by infection with tiny tapeworms of the genus *Echinocococcus*. The parasite usually originates from dogs and other livestock, and then transmitted to human through accidental ingestion of food or water contaminated with tapeworm eggs. Based on the types of the tapeworm, echinococcosis is classified as either cystic echinococcosis or alveolar echinococcosis. Infection with both types of echinococcosis can cause serious results to the patients and require compound treatment involving complex surgery and medical management (1–3).

Alveolar echinococcosis (AE) is an infrequent zoonosis caused by infection of *Echinococcus multilocularis* (4–6), and mostly common in the northern hemisphere with an incidence of 0.03–1.2/100,000 person per year worldwide (7, 8). As the worm cysts grow slowly in the human body, AE might be concealed for 5–15 years, and the natural history may mimic malignancy. If it is not diagnosed early, extensive surgery and prolonged drug therapy are usually required. Though AE mainly involves the liver, it infrequently spreads to other organs including the spleen, lungs or brain (9). Involvement of vertebrae is extremely rare, with few cases reported in the past decade, making it susceptible to misdiagnosis (10–12).

Current diagnosis of AE mainly relies on CT or MRI scan and blood testing for tapeworm antibodies against *Echinococcus multilocularis*, but limited sensitivity and specificity is noted in extrahepatic AE. Recent advances in metagenomic next-generation sequencing (mNGS) technologies have allowed clinicians to address complicated and concealed infections. Herein, we report a suspected tuberculosis infection case with unexplained back pain and unhealed back wound, with a final diagnosis of vertebral AE by mNGS.

## Case presentation

**Figure 1:**
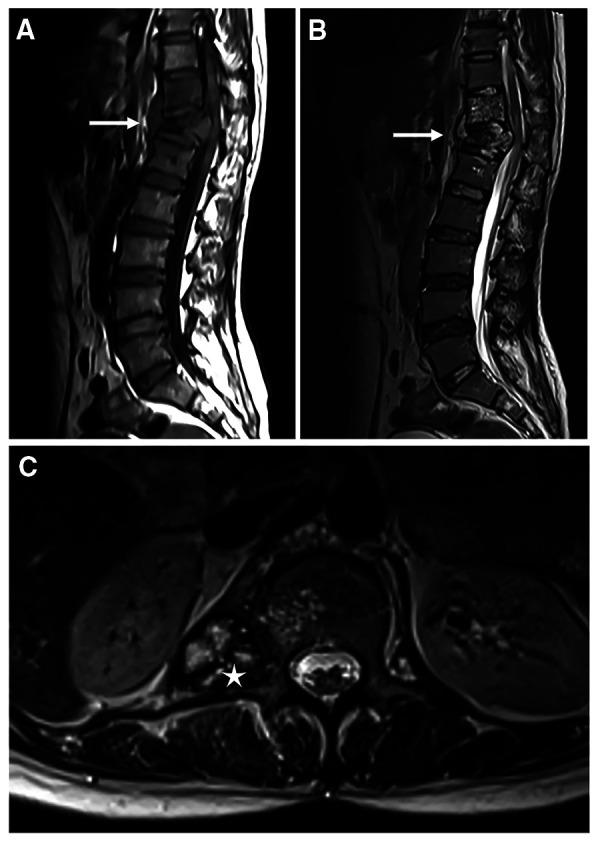
Magnetic resonance imaging of the thoracolumbar spine. T1-weighted image (**A**) and T2-weighted STIR image (**B**) of the destructed thoracolumbar spine in sagittal orientation, white arrows indicate destruction of vertebrae; Cystic mass (white asterisk) is found in the T2-weighted image in cross-sectional orientation (**C**).

**Figure 2:**
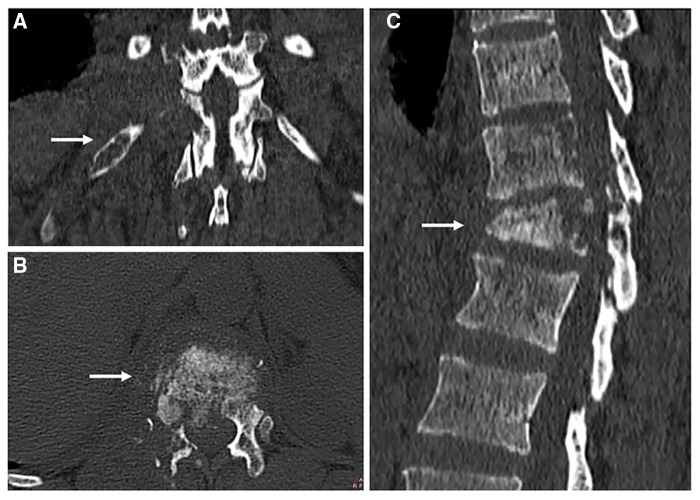
Computed tomography scans of the thoracolumbar spine. Local magnification of cross-sectional (**A**), coronal (**B**) and sagittal images. White arrows indicate destruction of vertebrae (**A,C**), and destruction of the 12th rib (**B**).

A 44-year-old woman presented with persistent neck and back pain for over one year. On initial admission one year ago, Magnetic resonance imaging (MRI) suggested destruction of the thoracic vertebrae (T11 and T12) (Figures 1A,B), and computed tomography (CT) scans revealed destruction of the right 12th rib (Figure 2). Radiology consult suggested the findings resembled spinal tuberculosis. The patient underwent surgical removal of focal necrosis in the thoracic spine and thoracolumbar vertebroplasty. Though biopsy of the necrotic tissue did not reveal *Mycobacterium tuberculosis*, the patient received empirical antituberculosis therapy in the setting of her disease manifestations and no history of Bacillus Calmette–Guérin (BCG) vaccine. During follow-up, an abscess developed adjacent to the back wound and the patient underwent an open debridement of abscess six months after the surgery.

The wound of the debridement healed, but repetitive neck and back pain struck the patients once again one month before the current admission, accompanied by numbness of bilateral upper limbs and neck immobility. Physical examination revealed a 20 cm healed surgical wound in her back. Tenderness and percussion pain were noted in the interspinous and spinous process of each cervical cone. Muscle tone was found in the bilateral trapezius, rhomboid and sternocleidomastoid muscles. MRI showed multiple cystic masses in the craniocervical junction region and effusion around the lumbar vertebrae (Figure 1C). Nothing special was noted in the abdominal contrast enhanced CT of the patient (Supplementary Figure S1). Blood tests suggested mildly elevated white blood cells with normal eosinophil count. Fecal examination suggested mildly positive occult blood. Multiple attempts were made to detect tuberculosis and other pathogens through both puncture of the effusion and cerebrospinal fluid, through culture and histopathological examination, but the results were all negative.

Though spinal tuberculosis was the initial leading diagnosis in the case of this patient, the negative results from multiple biopsies led us question our assumptions and conduct further testing. Therefore, we adopted mNGS to further screen for potential pathogens in this case, and the results revealed high reads count and coverage rate for *Echinococcus multilocularis*, which was further verified by PCR and Sanger sequencing.

**Figure 3:**
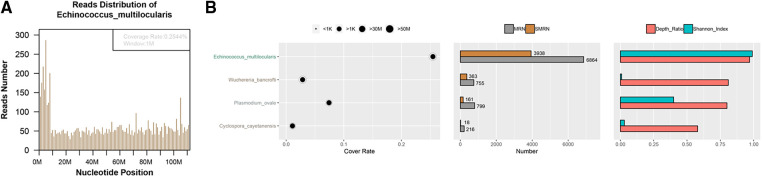
Investigation and validation results of metagenomic next-generation sequencing. Mapping of the detected reads from the specimen cover the genome of Echinococcus multilocularis (**A**); Echinococcus multilocularis was identified as the most predominant pathogen constituting 99.74% (3,938 reads) of the total sequence reads (**B**).

To investigate the underlying reason of abscess formation, a DNBseq™ mNGS detection of the punctured effusion was performed. The detailed protocol is established as follows. First, 7.2 *μ*l lysozyme was added into 0.6 ml effusion sample for wall-breaking reaction. Then we added the above solution to a 1.5 ml microcentrifuge tube with 250 *μ*l 0.5 mm glass beads and placed into the FastPrep-24™ 5G bead beating grinder and lysis system for vigorous agitation. Next, we separated 0.3 ml sample into a new 1.5 ml microcentrifuge tube and used the TIANamp Micro DNA Kit (DP316, TIANGEN BIOTECH) to extract DNA. DNA libraries were constructed through DNA-fragmentation, end-repair, adapter-ligation and PCR amplification. Agilent 2,100 was used for quality control of the DNA libraries. Quality qualified libraries were pooled, DNA Nanoball was made and sequenced by MGISEQ-2000 platform (13). High-quality sequencing data were generated by removing low-quality reads, followed by computational subtraction of human host sequences mapped to the human reference genome (hg19) using Burrows-Wheeler Alignment (14). The remaining data were classified by simultaneously aligning to the Pathogens metagenomics Database, consisting of bacteria, fungi, viruses and parasites. The classification reference databases were downloaded from NCBI (ftp://ftp.ncbi.nlm.nih.gov/genomes/). The total number of reads detected was 176,712,306, of which 73,044 aligned to microorganisms. Among them, the detected reads from the specimen mapped well with the *Echinococcus multilocularis* genome (Figure 3A). As for the constitution, *Echinococcus multilocularis* was identified as the most predominant pathogen taking up to 99.74% (3,938 reads) of the total sequence reads (Figure 3B).

**Figure 4:**
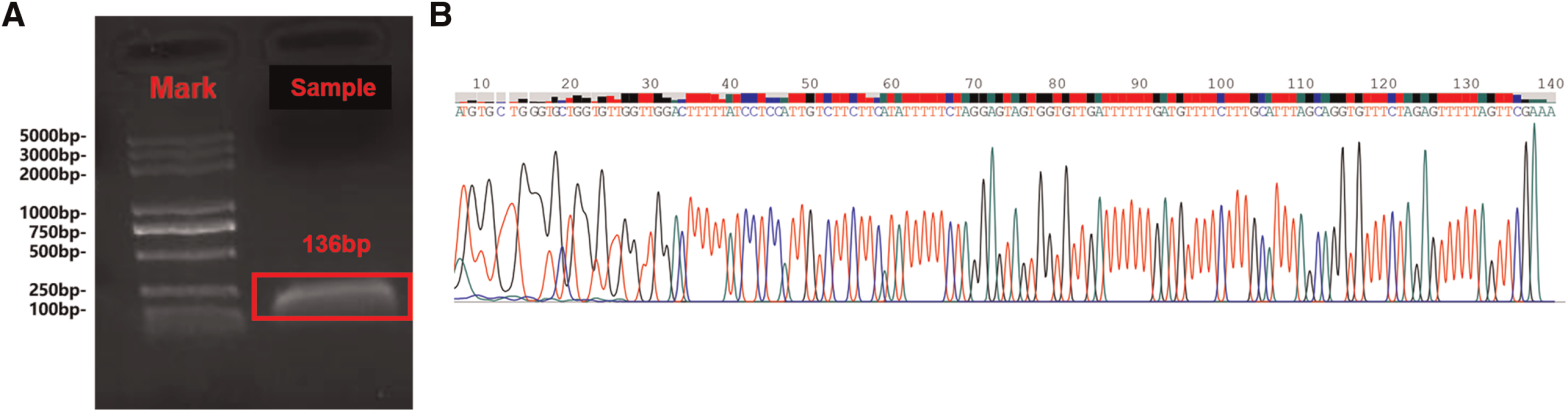
The Sanger sequencing results are consistent with sequence alignment of the tapeworm cox1 gene. Gel electrophoresis of PCR products (**A**); Sanger sequencing results (**B**).

To verify the mNGS result, we performed Sanger sequencing on the extracted sample DNA. According to the mNGS result, we designed a pair of PCR primers (EfF: 5'-GATTCCTTCTTTAGTTTTGTTGTTGATTAG-3' and EfR: 5'-CGAACTAAAAACTCTAGAAACACCTGCT-3') to specifically amplify the *COX1* gene of *Echinococcus multilocularis* (GenBankTM accession number: AB813188). The sequencing results are consistent with the sequence alignment of the tapeworm cox1 gene, which further confirmed the mNGS results (Figure 4).

After diagnosis of vertebral AE, the antituberculosis therapy was ceased and a continuous Benzimidazole treatment with Albendazole 400 mg twice per day was imitated immediately. The local symptom of the patient improved and was waiting for the next period of follow-up. No adverse events were noted one month after the Benzimidazole therapy. Our case was reported based on the CARE reporting standard (Supplementary Table S1). The whole timeline of the patients was displayed in Supplementary Figure S2.

## Discussion

### AE background

AE is primary a disease of animals, humans are accidental intermediate hosts, with liver most commonly affected (15). Despite its late onset and various symptoms, the diagnosis of the classic pattern of the disease, i.e. intrahepatic AE, is well established (16). However, extrahepatic AE is always difficult to diagnose, or might even be misdiagnosed due to its rare incidence, lingering course and diverse clinical manifestations. The more common sites of involvement of extrahepatic echinococcosis are spleen, lungs, or brain, while vertebrae are scarcely reported. To the best of our knowledge, this is the first case reporting a vertebral AE diagnosed by mNGS, which may provide a new approach of differentiating vertebral AE with other potential pathogens.

### Differential diagnosis of spinal AE and tuberculosis

In this case, the patient was initially suspected of spinal tuberculosis in a local hospital based on her clinical course and imaging results. Though spinal involvement represents less than 1% of overall tuberculosis, spinal tuberculosis is one of the most destructive and common forms of musculoskeletal tuberculosis, accounting for approximately 50% of musculoskeletal cases (17). Spinal tuberculosis usually manifests as local pain, tenderness, stiffness of the muscles, and a cold abscess, as well as a prominent spinal deformity, which is consistent with the onset and natural course of the presented case. Likewise, spinal AE can also generate the above symptoms, with pain as the most frequent symptom and neurological deficits as the second most common symptom (11). Due to similar clinical presentations, the differential diagnoses of vertebral AE mainly include vertebral tuberculosis (Pott's disease), bacterial or fungal abscess, and malignant or benign tumors (12).

In the recent decade, a two-part comprehensive systematic review of spinal cystic echinococcosis (CE) has helped clinicians gain a deeper understanding of this species (18), but given the different nature of the two tapeworms, the facts and implications of CE might not be equally applicable to spinal AE. In the few previously reported cases, vertebral AE was commonly diagnosed by biopsy, PCR, and serology (11). However, biopsy may have a low sensitivity depending on sampling density and heterogeneity, while serology testing might not be able to distinguish co-infection or immunological cross-reaction (19). The present case emphasized the importance of questioning the initial diagnosis when the course of disease mismatched with our assumptions (15), more effective and precise diagnostic methods should be used.

Recent advances in mNGS technologies have allowed clinicians to address complicated and concealed infections. This case represented an example of distinguishing a suspected tuberculosis infection with a final diagnosis of vertebral AE following mNGS.

### Clinical application of mNGS

With broad coverage, fast detection speed, and high accuracy, mNGS represents a new, less biased approach that can detect thousands to millions of nucleotides fragments at once (20). Compared to other traditional genetic diagnostic methods, such as PCR, mNGS can detect a wide range of hard-to-culture, atypical and rare pathogens without prior target knowledge (21). However, the relatively high cost, complicated procedure and strict protocol for preventing specimen contamination limit the wider application of NGS, which requires a professional and well-trained laboratory team. Nevertheless, mGNS has gained high sensitivity in complex pathogen identification, e.g. Mycobacterium tuberculosis, viruses, anaerobes and fungi, especially in cases exposed to prior antibiotics (20). This case also provided a novel perspective for distinguishing suspected coinfections and diagnosis of rare parasitic zoonosis by mGNS. As is pointed out by Han and colleagues (22), mNGS is transforming the landscape of clinical microbiology laboratories, the appropriate utilization will help physicians and microbiologists better distinguish complex infections.

### Treatment of extrahepatic AE

The management of extrahepatic AE mainly involves surgical resection and antiparasitic drug usage. As our case already had a past surgical history, we first started Benzimidazole treatment with Albendazole as the main antiparasitic agent. In addition, Akbulut S also reported that amphotericin B can also be used as an effective drug to treat alveolar echinococcal disease, especially in those patients with hepatic toxicity after benzimidazole treatment or treatment resistant cases (23).

## Conclusion

To the best of our knowledge, this is the first case differentiating a vertebral AE from a suspected spinal tuberculosis by mNGS, which may provide a new insight of fast and accurate diagnosis of clinical occult infection. The application of mNGS technology could assist clinicians to screen, detect and distinguish potential pathogens rapidly and efficiently, especially under complicated circumstances.

## Data Availability

Publicly available datasets were analyzed in this study. This data can be found here: The de-identified data and information from this study can be requested by email of the corresponding author.

## Supplementary material

Supplementary Figure S1 Contrast-enhanced computed tomography scans of the abdomen. No special lesions was observed in the liver and spleen.

Supplementary Figure S2 Timeline showing the clinical course of the patient.
