# Supplementary figures and images for: Case Report: Diagnosis of vertebral alveolar echinococcosis upon next-generation sequencing in a suspected tuberculosis
Source: Front Surg. 2022 Sep 29;9:984640. doi: 10.3389/fsurg.2022.984640 (PMC9556986; doi:10.3389/fsurg.2022.984640)

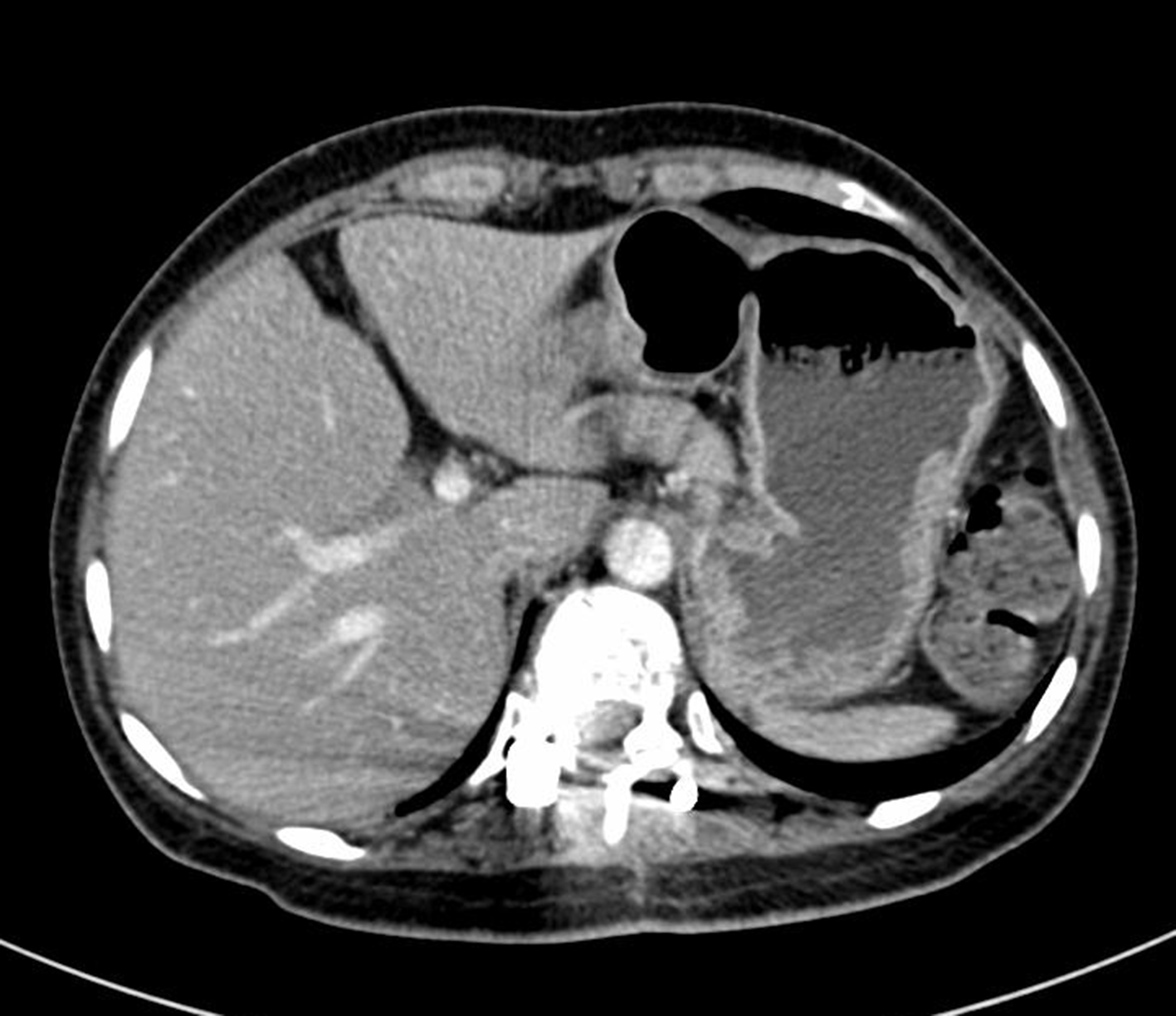

Supplement: Supplementary Figure S1 — Contrast-enhanced computed tomography scans of the abdomen. No special lesions was observed in the liver and spleen. [file Image1.tif]

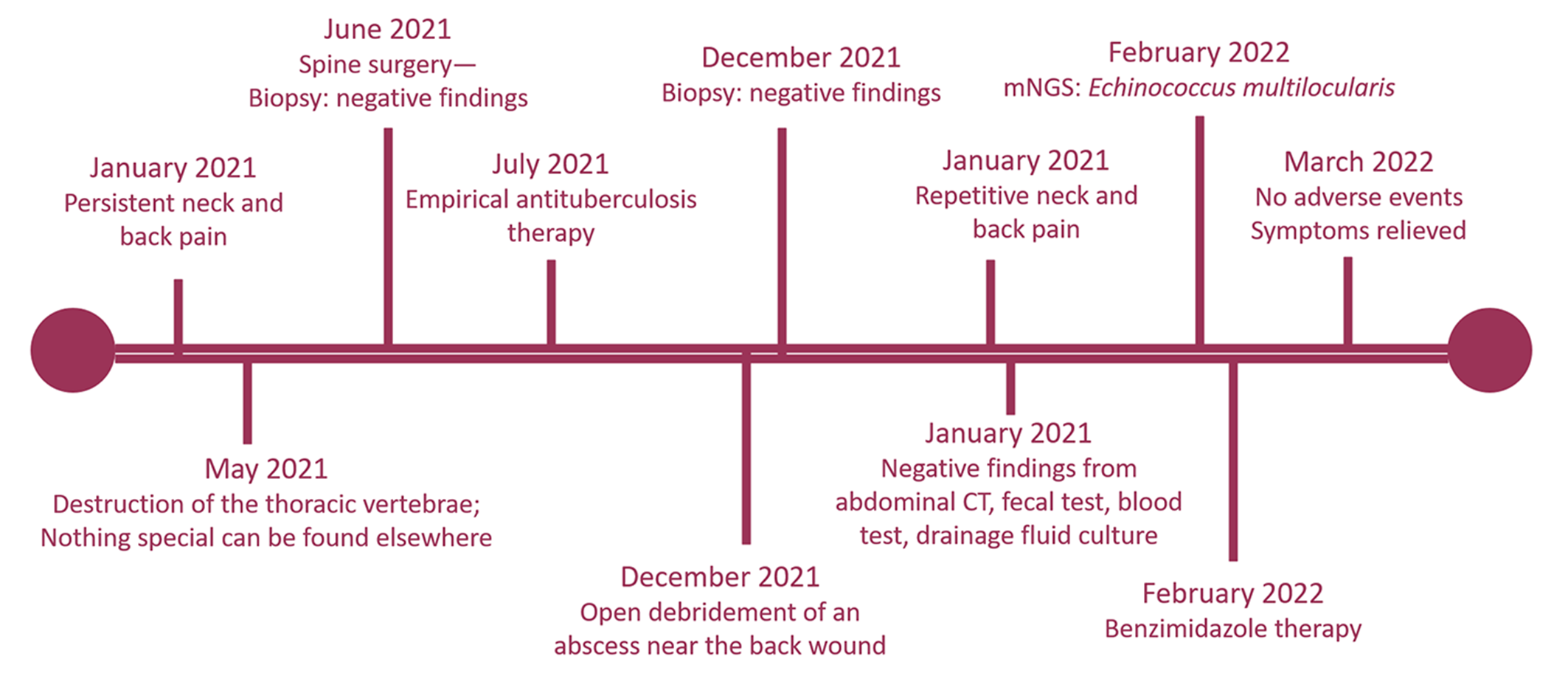

Supplement: Supplementary Figure S2 — Timeline showing the clinical course of the patient. [file Image2.tif]
