# Supplementary material for: Case Report: Diagnosis of vertebral alveolar echinococcosis upon next-generation sequencing in a suspected tuberculosis
Source: Front Surg. 2022 Sep 29;9:984640. doi: 10.3389/fsurg.2022.984640 (PMC9556986; doi:10.3389/fsurg.2022.984640)
Supplement: Supplementary file 3 [file Table1.docx]

***Supplementary Material***

**Supplementary Table 1. CARE checklist**


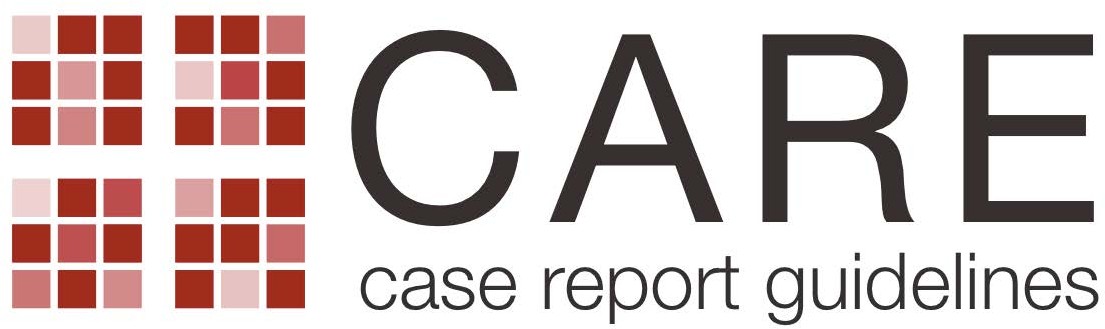
CARE Checklist of information to include when writing a case report
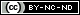


**Topic Item Checklist item description Reported on Line**

**Title 1** The diagnosis or intervention of primary focus followed by the words “case report” 1

**Key Words 2** 2 to 5 key words that identify diagnoses or interventions in this case report, including "case report" 20-21

# Abstract

**(no references)**

**3a** Introduction: What is unique about this case and what does it add to the scientific literature? 27-29

**3b** Main symptoms and/or important clinical findings 30-34

**3c** The main diagnoses, therapeutic interventions, and outcomes 34-36

**3d** Conclusion—What is the main “take-away” lesson(s) from this case? 37-40

**Introduction 4** One or two paragraphs summarizing why this case is unique (**may include references**) 52-57

**Patient Information 5a** De-identified patient specific information 59

**5b** Primary concerns and symptoms of the patient 59-60

**5c** Medical, family, and psycho-social history including relevant genetic information 63-66

**5d** Relevant past interventions with outcomes 63-71

# Clinical Findings

**Timeline**

**Diagnostic Assessment**

**Therapeutic Intervention**

**Follow-up and Outcomes**

1. Describe significant physical examination (PE) and important clinical findings 69-73
2. Historical and current information from this episode of care organized as a timeline 59-78

**8a** Diagnostic testing (such as PE, laboratory testing, imaging, surveys). 75-78

**8b** Diagnostic challenges (such as access to testing, financial, or cultural) 79-80

**8c** Diagnosis (including other diagnoses considered) 81-83

**8d** Prognosis (such as staging in oncology) where applicable NA

**9a** Types of therapeutic intervention (such as pharmacologic, surgical, preventive, self-care) 65-66

**9b** Administration of therapeutic intervention (such as dosage, strength, duration) 110-111

**9c** Changes in therapeutic intervention (with rationale) 110-111

**10a** Clinician and patient-assessed outcomes (if available) 112

**10b** Important follow-up diagnostic and other test results NA

**10c** Intervention adherence and tolerability (How was this assessed?) NA

**10d** Adverse and unanticipated events 113

**Discussion 11a** A scientific discussion of the strengths AND limitations associated with this case report 147-156

**11b** Discussion of the relevant medical literature **with references** 116-156

**11c** The scientific rationale for any conclusions (including assessment of possible causes) 158-162

**11d** The primary “take-away” lessons of this case report (without references) in a one paragraph conclusion 158-162

**Patient Perspective 12** The patient should share their perspective in one to two paragraphs on the treatment(s) they received 160-162

**Informed Consent 13** Did the patient give informed consent? Please provide if requested . . . . . . . . . . . . . . . . . . . . . . . . . . . . . . . . . . . . . . **Yes√ No**

**
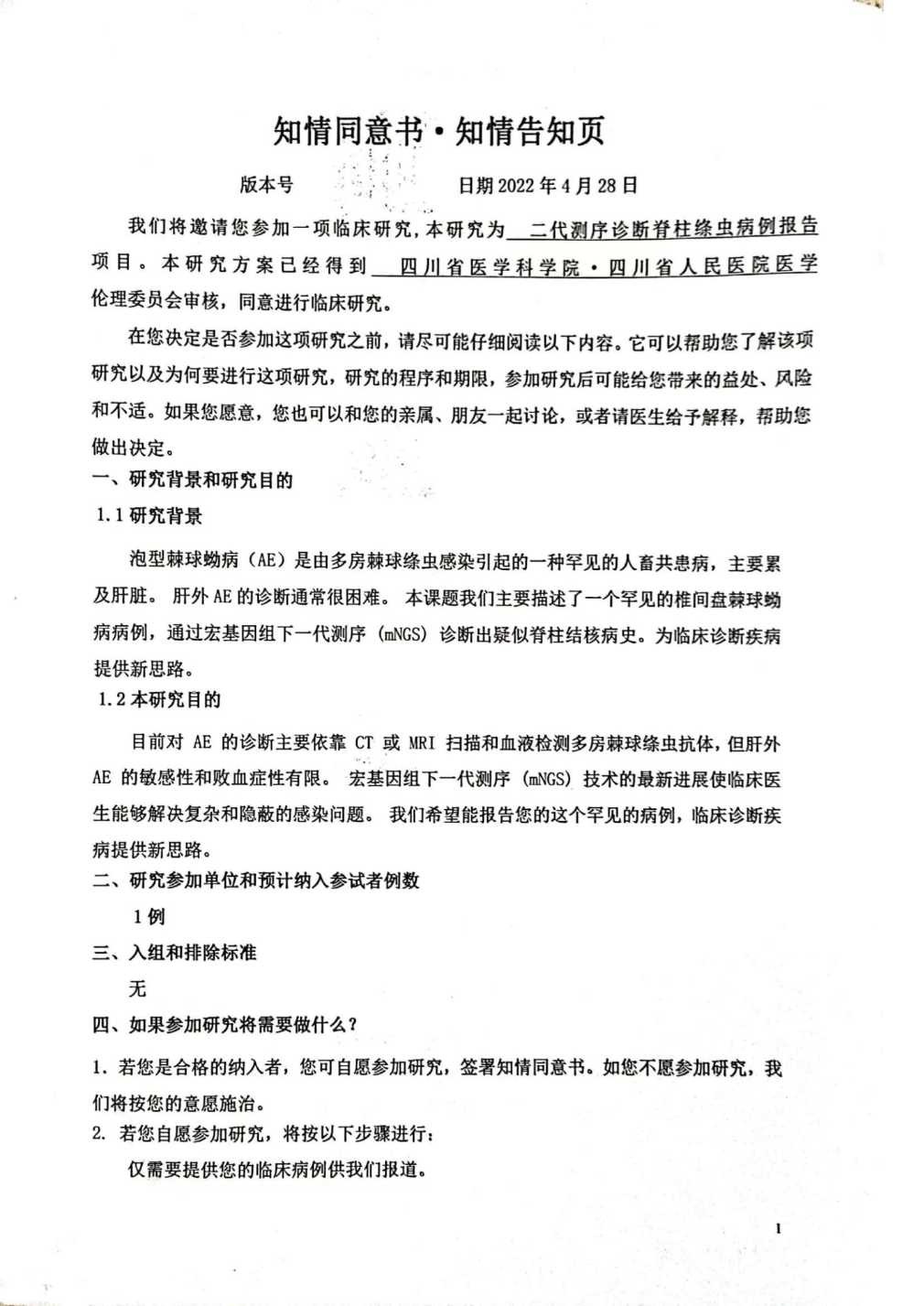
Supplementary file 1. Signed informed consent from the patient**
